# Expression of immune checkpoints on circulating tumor cells in men with metastatic prostate cancer

**DOI:** 10.1186/s40364-021-00267-y

**Published:** 2021-02-18

**Authors:** Tian Zhang, Anika Agarwal, R. Garland Almquist, Daniella Runyambo, Sally Park, Elizabeth Bronson, Rengasamy Boominathan, Chandra Rao, Monika Anand, Taofik Oyekunle, Patrick Healy, Megan A. McNamara, Kathryn Ware, Jason A. Somarelli, Daniel J. George, Andrew J. Armstrong

**Affiliations:** 1 Division of Medical Oncology, Department of Medicine, Duke Cancer Institute, Duke University, DUMC 103861, Durham, NC 27710 UK; 2 Duke Cancer Institute Center for Prostate and Urologic Cancers, Durham, NC UK; 3 Janssen Pharmaceuticals Research & Development, Spring House, PA USA; 4 Department of Biostatistics and Bioinformatics, Duke University, Durham, NC UK; 5 Department of Pharmacology and Cancer Biology, Duke University, Durham, NC UK

**Keywords:** Circulating tumor cells, Metastatic prostate cancer, PD-L1, PD-L2, CTLA-4

## Abstract

**Background:**

A subset of men with metastatic prostate cancer (mPC) responds to immune checkpoint inhibitors, and there is an unmet need to predict those most likely to benefit. We characterized circulating tumor cells (CTCs) for expression of immune checkpoint ligands in men with mPC as a non-invasive biomarker of immune evasion and immunotherapy benefit.

**Methods:**

Three cohorts of patients were enrolled: 1) men with mCRPC starting abiraterone acetate/prednisone or enzalutamide (pre-ARSI), 2) men with mCRPC who were progressing on enzalutamide or abiraterone acetate/prednisone (post-ARSI), and 3) men with newly diagnosed metastatic hormone sensitive prostate cancer (mHSPC) starting androgen deprivation therapy. CTCs were captured using the CellSearch® system and stained for PD-L1, PD-L2, B7-H3, and CTLA-4 at baseline, on treatment, and disease progression. Summary statistics on mean CTCs per cohort, as well as rates of ligand positivity were used to analyze CTCs by cohort and by timepoint.

**Results:**

Men in all cohorts and timepoints had prevalent CTC B7-H3 expression (> 80%). We found evidence for CTC PD-L1 expression across disease states, in which > 1 positive CTC or > 50% of CTCs were positive for PD-L1 in 40 and 30% of men with mHSPC, respectively, 60 and 20% of men with mCRPC pre-ARSI, and 70 and 30% of men with mCRPC post-ARSI. CTC PD-L2 expression was present in 20–40% of men in each disease state, while CTC CTLA-4 expression was rare, present in 20% of men with mCRPC pre-ARSI and 10% of men with mCRPC post-ARSI or with mHSPC. CTC immune checkpoint expression was heterogeneous within/between men and across disease states.

**Conclusions:**

We have identified that CTCs from men with mPC heterogeneously express immune checkpoints B7-H3, PD-L1, PD-L2, and CTLA-4, and the detection of these immune checkpoints may enable monitoring on immunotherapy.

**Supplementary Information:**

The online version contains supplementary material available at 10.1186/s40364-021-00267-y.

## Introduction

Prostate cancer is the most common cancer in men, causing the second-highest number of cancer-related deaths per year [1]. Targeting androgen availability using androgen deprivation therapy (ADT) is initially effective; however, progression to metastatic, castration-resistant prostate cancer (mCRPC) ensues in many cases. The mCRPC phenotype is aggressive and often lethal, with limited effective therapies [2, 3]. The immunogenicity of mCRPC has been controversial, with many studies demonstrating an absence of infiltrating T-cells in the majority of human prostate cancer microenvironments, and thus prostate cancer has been referred to variably as an immune desert [4, 5].

As our understanding of tumor-immune interactions evolves [6, 7], efforts have been made to alter the adaptive immune response, manipulating effector T-cells by targeting their tightly-regulated system of activation and suppression signals. These efforts have led to significant advances in immunotherapy by blocking checkpoint receptors on T-cells (including cytotoxic T-lymphocyte antigen-4 [CTLA-4] and programmed cell death 1 [PD-1]) through a class of immunotherapy checkpoint inhibitors (ICIs). ICIs have improved survival outcomes for multiple metastatic solid tumors, but the clinical data for use in prostate cancer thus far have not shown significant efficacy [8–11]. As monotherapy in mCRPC, pembrolizumab has shown objective response rates based on RECIST 1.1 criteria of around 5%, disease control rate (stable disease or better) of up to 23% in men with bone-predominant disease, > 50% PSA decline rates of up to 8%, and median PFS of 2.1 months (in patient with visceral disease) and 3.7 months (in men with bone-predominant disease) [10]. In a recent trial of pembrolizumab with enzalutamide in 28 men, the primary endpoint of > 50% PSA decline was observed in 18%, and objective response rate was 25% (3 of 12 with measurable disease) [11].

One reason for the relatively poor response of men with mCRPC to ICIs is the limited understanding of the immune checkpoint targets in prostate cancer. Recent reports have implicated the innate immunity STING pathway [12], the checkpoint ligands PD-L1 and PD-L2, and B7H3 as potential immunotherapy targets for prostate cancer. Both PD-L1 expression on tumor cells and PD-1 expression on antigen presenting cells were up-regulated in mCRPC men resistant to enzalutamide [13]. PD-L1 has also been previously identified on circulating tumor cells (CTCs) in metastatic breast, prostate, colorectal, lung, and urothelial cancers [14, 15]. Gene expression profiling of prostatectomy specimens also showed that higher PD-L2 expression was prognostic, associated with worse biochemical recurrence, metastatic spread, and prostate cancer specific survival [16]. B7-H3 and CTLA-4 were selected as the other checkpoints to characterize in this study for their prevalence on prostate cancer cells and importance in the tumor-immune interactions, respectively. In particular, B7-H3 expression has been characterized as a prevalent checkpoint on primary prostate tumors [17], correlates with prostate cancer progression and poor outcomes [18, 19], and was unchanged in the tumor microenvironment after treatment with androgen receptor signaling inhibitors (ARSI) enzalutamide or abiraterone acetate plus prednisone [20].

Although these therapies are not effective across the entire continuum of mCRPC phenotypes, biomarker-selected populations of mCRPC men have shown some benefit from ICIs, including men with mismatch repair deficiency [21, 22], biallelic *CDK12* loss [23–25] and mutations in homologous recombination genes such as *BRCA2* [26, 27]. In addition to the responses from some underlying genotypic conditions, ICIs response may also be improved in the enzalutamide-resistant setting [13, 28, 29]. In enzalutamide-resistant disease, AR blockade inhibits the DNA damage repair processes, which may explain, in part, the increased responses to ICIs.

In addition to genomics-based markers of treatment response, several markers of immunogenicity in the tumor microenvironment have also been shown to predict for prostate cancer aggressiveness and resistance, which may hold promise for better understanding response and resistance to ICIs. Real-time biomarkers that reflect changes in the tumor microenvironment have the potential to improve disease monitoring and treatment. As a real-time biomarker, CTCs are disseminated cancer cells in circulation that are representative of metastatic, therapy-resistant disease [30–32]. CTCs can further reflect changes in the tumor microenvironment better than archival tissue specimens, and can provide peripheral monitoring of cell surface changes [33]. In prostate cancer, the CellSearch® platform has been used to quantify CTC burden in men with metastatic castration resistant disease and is FDA cleared as a prognostic biomarker of overall survival [34, 35]. Molecular and digital pathology profiling of CTC biomarkers has also validated the clinical utility of CTC characterization for identifying prognostic and predictive biomarkers of treatment response in a range of clinical settings, including the development of resistance to enzalutamide [36–38], abiraterone acetate plus prednisone [39], and taxanes [40–42]. We have previously described the capture of CTCs based on expression of the MET oncogene [43], and through genomic profiling of CTCs, have found that hormone therapy resistance can be due to AR mRNA splice variants, AR signaling loss [37] and the gain of neuroendocrine-like features [44]. Together, these studies demonstrate the power and utility of analyzing CTC biomarkers for both prognostic and predictive benefit.

In this study, we sought to capitalize on the unique benefits of CTC biomarker characterization to better understand the molecular landscape of immune checkpoint expression in prostate cancer. To this end, the main objective of this study was to describe the expression of PD-L1, PD-L2, B7-H3, and CTLA-4 on CTCs isolated from men with metastatic prostate cancer across different hormone sensitive and castration resistant disease states and over time during treatment and disease progression, for the purposes of future therapeutic targeting and prognostic biomarker studies of immune checkpoint inhibition in men with metastatic prostate cancer.

## Materials and methods

### Marker validation studies

**Fig. 1:**
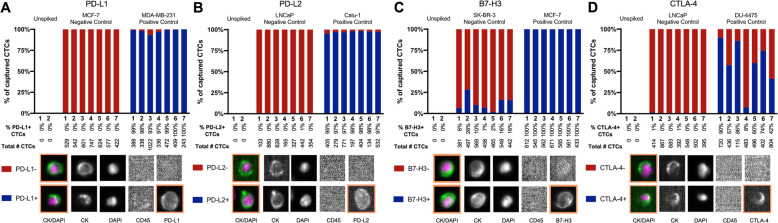
Healthy volunteer spiking experiments for (A) PD-L1, (B) PD-L2, (C) B7-H3, and (D) CTLA-4 checkpoint ligands on CTCs. Cell lines known to be positive or negative for expression of each immune checkpoint biomarker were spiked into healthy volunteer whole blood. Control samples were spiked with approximately 500 cells (variation due to random error). Each panel shows unspiked, negative and positive control cells captured from these spiking experiments, with total # of CTCs captured below. Panels of CTCs depicted below are the raw CTC output from Cellearch – from left to right, merged image of captured cell, cytokeratin (CK) stain, the DAPI nuclear stain, CD45 (leukocyte marker, negative in tumor cells), and the checkpoint ligand of interest

The sensitivity and specificity of EpCAM-based capture of CTCs and downstream immune checkpoint biomarker expression has been validated previously and applied here, with sensitivity/specificity of each fluorescent marker evaluated in control cancer cell lines (both positive and negative controls) [45]. For PD-L1, the breast cancer cell lines MDA-MB-231 was used as positive control and MCF7 was used as negative control. For PD-L2, the lung cancer cell line Calu-1 was used as positive control and LNCaP prostate cancer cell line was used as negative control. For B7-H3, the breast cancer cell lines MCF7 was used as positive control and SKBR3 was used as negative control. Finally for CTLA-4, the breast cancer cell line DU4475 was used as positive control and LNCaP was used as negative control. Commercially available antibodies conjugated to phycoerythrin and directed against PD-L1 (29E.2A3, Biolegend, San Diego, CA); PD-L2 (MIH18, Biolegend, San Diego, CA); B7-H3 (MIH42, Biolegend, San Diego, CA), and CTLA-4 (BNI3, BD Bioscience, San Diego, CA) were used to validate positive and negative control cell lines, followed by healthy volunteer spiking studies. Each healthy volunteer whole blood sample was spiked with approximately 500 cells from a positive or negative control cell line for each immune checkpoint ligand, and these spiking studies were repeated approximately every two months for quality control of these immune checkpoints’ characterization throughout the study (Fig. 1). Healthy volunteers were recruited under a Duke IRB approved protocol (Pro00027680) and provided informed consent.

### Study design and patient characteristics

This prospective correlative study enrolled men with metastatic adenocarcinoma of the prostate being treated with standard of care therapies. The study was approved through the Duke Institutional Review Board and registered (NCT02456571). Men were recruited from the Duke Cancer Institute. Men were eligible for enrollment across each of the following three metastatic disease states: 1) cohort A: men with mCRPC starting abiraterone acetate/prednisone or enzalutamide (pre-ARSI), 2) cohort B: men with mCRPC who were progressing on enzalutamide or abiraterone acetate/prednisone (post-ARSI), and 3) cohort C: men with newly diagnosed metastatic hormone sensitive prostate cancer (mHSPC) starting androgen deprivation therapy (ADT) or ADT/docetaxel chemotherapy. Men were recruited under a Duke IRB approved protocol and provided informed consent. This pilot study was designed with the hypotheses that at least 2 patients out of 10 in each cohort will have either PD-L1, PD-L2, or CTLA-4 expressed on CTCs (20% prevalence) and for B7-H3, that at least 5 patients out of 10 will have B7-H3 positivity on CTCs.

Men eligible for inclusion were ≥ 18 years of age with histologically confirmed diagnosis of prostate adenocarcinoma. Eligible men also had clinical or radiographic evidence of progressive metastatic disease, with progression defined as a rising PSA, new metastatic lesions (bone or soft tissue), or radiographic evidence of tumor growth on CT or MRI. Men with adenocarcinoma of the prostate in cohorts A and B (with mCRPC pre- or post-ARSI) were required to have castrate levels of testosterone (≤50 ng/dl). Men in cohort C (mHSPC) with a history of hypogonadism were excluded. Men who had treatment with an anthracycline (including mitoxantrone) within 1 week of CTC collection were also excluded, as anthracyclines cause auto-fluorescence of cells. Additionally, consented men were considered screen fails for the study if no CTCs were detected at the baseline blood draw and these men were replaced.

### Sample collection

Peripheral blood was collected aseptically by venipuncture or from a venous port into CellSave® Preservative tubes (Menarini Silicon Biosystems, Huntingdon Valley, PA), approximately 7.5 mL per tube. Samples were collected at baseline prior to start of treatment, week 12, and treatment progression. Four separate CellSave® tubes were collected at each timepoint to assess for the 4 different immune checkpoint ligands of interest. Blood samples were stored or transported in CellSave® Preservative Tubes for up to 72 h at room temperature prior to processing.

### Circulating tumor cell (CTC) detection and capture

The CellSearch® technology (Menarini Silicon Biosystems, Huntingdon Valley, PA) is the only FDA-approved method for CTC capture, using anti-epithelial cell adhesion molecule (EpCAM) ferrofluid to capture CTCs. A commercially available CellSearch® CXC kit contains the reagents and supplies for this EpCAM-based capture of CTCs from whole blood, followed by confirmatory immunofluorescent staining of cytokeratin (CK), 4′,6-diamidino-2-phenylindole (DAPI), and CD45. CXC-type kits were constructed for EpCAM-based capture of CTCs and immune checkpoint characterization. In these CXC-type kits, CK is assessed with FITC while PE is used for the second channel immune checkpoint biomarker of interest (PD-L1 [Ventana SP142], PD-L2 [BioLegend MIH18], B7-H3 [BioLegend MIH42], and CTLA-4 [BioLegend BNI3]).

After immunomagnetic capture and enhancement, fluorescent reagents are added for identification and enumeration of the target cells. The processed reagent/sample mixture is dispensed by the CellTracks® AutoPrep System into a cartridge that is inserted into a MagNest® device. The strong magnetic field of the MagNest® device attracts the magnetically labeled epithelial cells to the surface of the cartridge. The CellTracks® Analyzer II is a semi-automated fluorescence microscope used to enumerate fluorescently labeled cells that are immunomagnetically selected and aligned. The CellTracks® Analyzer II scans the entire surface of the cartridge with a series of fluorescence filters that are defined for a given assay. Cell images from each filter are compiled and presented in a gallery format for final cell classification for identification and enumeration of CTCs. CTCs were defined as CK positive, DAPI positive nucleated cells lacking CD45*.* Events were first manually identified as CTCs and then scored if the immune checkpoint ligand was expressed. This procedure has been standardized and published using specimens derived from men with advanced prostate cancer [46]. Summary statistics on mean CTCs per cohort, as well as rates of ligand positivity were used to analyze CTCs by cohort and by timepoint.

## Results

### Healthy volunteer spiking assays

Healthy volunteer peripheral blood had no detectable CTCs (Fig. 1). To further validate the ability to detect cells expressing immune checkpoints in blood, peripheral blood samples from healthy volunteers were spiked with control cell lines as described above. For the checkpoint ligands PD-L1, PD-L2, B7-H3, and CTLA-4, positive control cell lines were identified as MDA-MB-231, Calu-1, MCF-7, and DU-4475, respectively; while negative control cell lines were identified as MCF-7, LNCaP, SK-BR-3, and LNCAP, respectively. Positive control cell lines ranged from mean 60% expression (DU-4475 cells for CTLA-4 expression) to mean 100% expression (MCF7 cells for B7-H3 expression). Negative control cell lines had range of mean 0% expression (MCF7 cells for PD-L1 expression) to mean of 0.3% expression (LNCaP cells for CTLA-4 expression) (Fig. 1).

### Patient characteristics

**Fig. 2:**
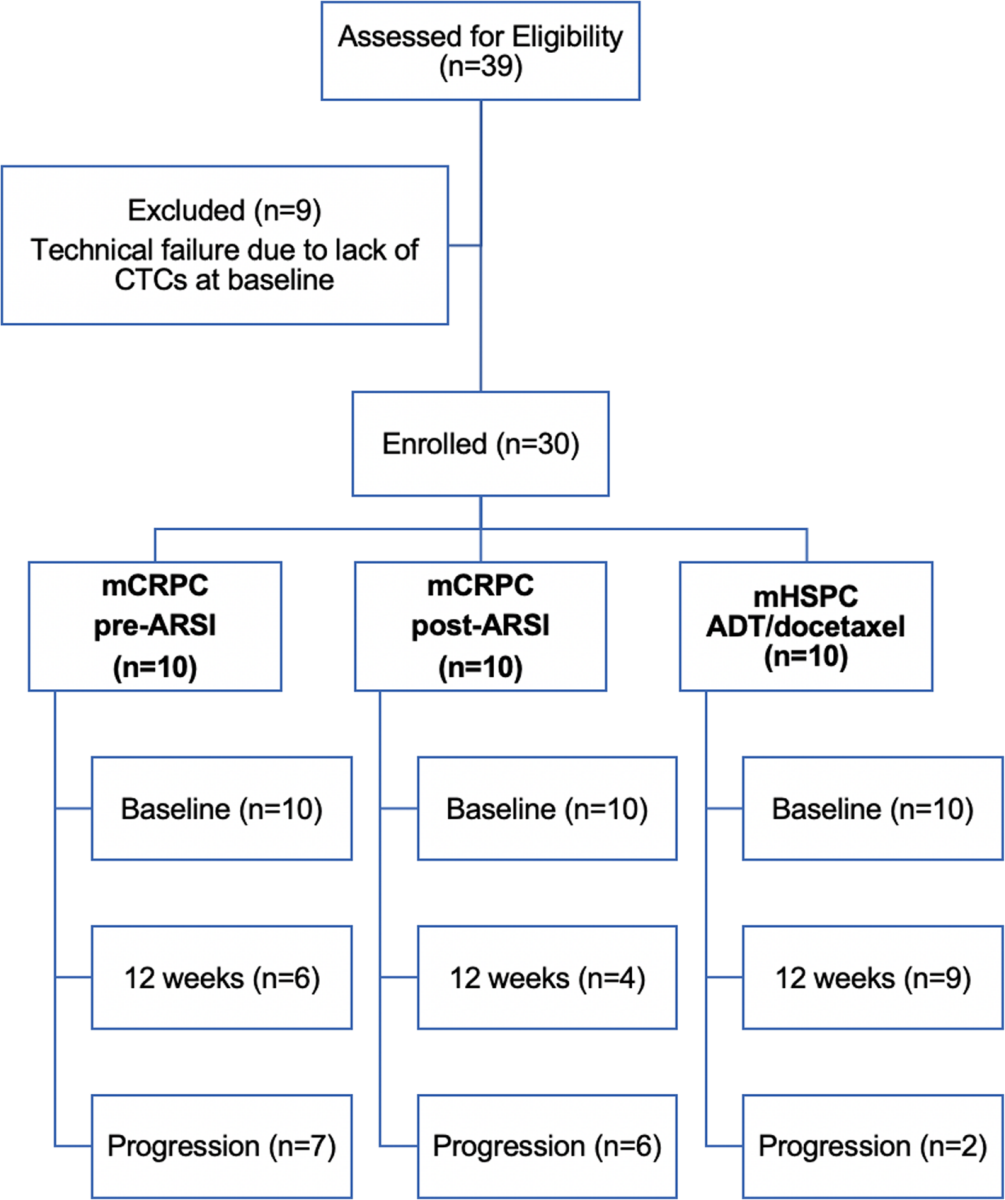
CONSORT diagram of men with metastatic prostate cancer with circulating tumor cells examined in this study. Cohort A: men with mCRPC starting abiraterone acetate/prednisone or enzalutamide (pre-ARSI), Cohort B: men with mCRPC who were progressing on enzalutamide or abiraterone acetate/prednisone (post-ARSI), and Cohort C: men with newly diagnosed metastatic hormone sensitive prostate cancer (mHSPC) starting androgen deprivation therapy (ADT) or ADT/docetaxel chemotherapy

Between November 28, 2016 and November 15, 2018, a total of 39 men were consented and assessed for eligibility. Nine men were replaced and considered unevaluable due to a lack of CTCs at baseline. Peripheral blood was collected from 30 men for CTC characterization, with 10 in each of the three cohorts as described above (CONSORT diagram shown in Fig. 2). In the mCRPC pre-ARSI cohort, median age of men was 74 years, generally had bone and lymph node predominant metastases with only 2 men with visceral metastases, presented with low baseline PSA (median 7 ng/mL) and only 30% with elevated LDH levels (median 83 U/L). In the mCRPC post-ARSI cohort, median age of men was 72 years, also had bone and lymph node predominant metastases and with only 2 men with visceral metastases, presented with median baseline PSA 27 ng/mL and 70% had elevated LDH levels (median 224 U/L). For the mHSPC cohort, median age was 72 years, all with bone/lymph node metastases (none with visceral metastases), men had higher PSA levels (median 90 ng/mL, range up to 1427 ng/mL), and 40% had elevated LDH levels (median 193 U/L) (Table 1).

**Table 1:** Baseline patient characteristics in three cohorts of metastatic prostate cancer

Baseline Characteristic	mCRPC pre-ARSI (***n*** = 10)	mCRPC post-ARSI (n = 10)	mHSPC starting ADT (n = 10)	Total (***n*** = 30)
**Age**				
**Median (range)**	**74 (51–83)**	**72 (53–88)**	**72 (55–82)**	**72 (51–88)**
**Race**				
**White**	**9 (90%)**	**9 (90%)**	**8 (80%)**	**26 (87%)**
**Black or African American**	**0 (0%)**	**0 (0%)**	**2 (20%)**	**2 (7%)**
**Other**	**1 (10%)**	**1 (10%)**	**0 (0%)**	**2 (6%)**
**PSA (ng/mL)**				
**Median (range)**	**7 (2–1327)**	**27 (5–126)**	**90 (4.3–1427)**	**32 (2–1427)**
**Hemoglobin (g/dL)**				
**Median (range)**	**12.2 (7.9–14.3)**	**12.2 (9.4–14.1)**	**13.6 (8.5–16.2)**	**12.6 (7.9–16.2)**
**< 13.7 g/dL (LLN)**	**9 (90%)**	**8 (80%)**	**5 (50%)**	**22 (73%)**
**LDH (U/L)**				
**Median (range)**	**238 (142–517)**	**224 (117–330)**	**193 (142–230)**	**224 (117–517)**
**> 100 U/L (ULN)**	**3 (30%)**	**7 (70%)**	**4 (40%)**	**14 (47%)**
**Alkaline phosphatase (U/L)**				
**Median (range)**	**83 (44–877)**	**87 (41–207)**	**111 (43–1378)**	**96 (41–1378)**
**> 110 U/L (ULN)**	**4 (40%)**	**3 (30%)**	**5 (50%)**	**12 (40%)**
**Prior Chemotherapy**				
**Cabazitaxel**	**0 (0%)**	**2 (20%)**	**0 (0%)**	**2 (7%)**
**Docetaxel**	**3 (30%)**	**5 (50%)**	**0 (0%)**	**8 (27%)**
**Metastatic Site**				
**Bone**	**8 (80%)**	**10 (100%)**	**9 (90%)**	**27 (90%)**
**Lymph Nodes (Inside Pelvis)**	**5 (50%)**	**3 (30%)**	**7 (70%)**	**15 (50%)**
**Lymph Node (Outside Pelvis)**	**4 (40%)**	**4 (40%)**	**4 (40%)**	**12 (40%)**
**Liver**	**0 (0%)**	**1 (10%)**	**0 (0%)**	**1 (3%)**
**Lung**	**2 (20%)**	**1 (10%)**	**0 (0%)**	**3 (10%)**
**Gleason Grade, Score (sum)**				
**<** **6**	**1 (10%)**	**1 (10%)**	**1 (10%)**	**3 (10%)**
**7**	**3 (30%)**	**3 (30%)**	**1 (10%)**	**7 (23%)**
**8–10**	**5 (50%)**	**6 (10%)**	**6 (60%)**	**17 (57%)**
**Missing**	**1 (10%)**	**0 (0%)**	**2 (20%)**	**3 (10%)**

At baseline, all men had detectable CTCs in at least one out of four blood samples and men without CTCs at baseline were replaced. Some men, however, did not have detectable CTCs at 12 weeks on treatment or at disease progression. Additionally, at a given timepoint, CTCs may have been detected in some samples, but not all four. As a result, the number of samples analyzed for each biomarker varied between timepoints and cohorts (see detailed biomarker CONSORT diagram in **Supplemental Fig.** **1**).

At the time of the data lock, with a median follow up of 21 months, 15 men showed evidence of disease progression (seven men in mCRPC pre-ARSI, six men in mCRPC post-ARSI, and two men in mHSPC) (Fig. 2).

### The number of detectable CTCs differs between men and cohorts

At baseline, the number of EpCAM-positive CTCs differed within and between cohorts. The median number of CTCs detected per 7.5 mL peripheral blood was 10 (range 0.3–181) in men with mCRPC pre-ARSI, five (range 0.8–291) in men with mCRPC post-ARSI, and three (range 1.3–73) in men with mHSPC (Table 2). About half of the men in all cohorts had at least five detectable CTCs; 50% of men with mCRPC pre-ARSI, 50% of men with mCRPC post-ARSI, and 40% of men with mHSPC.

**Table 2:** CTCs per cohort and by time point, including CTC immune checkpoint biomarker expression for baseline

	mCRPC pre-ARSI	mCRPC post-ARSI	mHSPC starting ADT	Total
**EPCAM CTC Number per 7.5 mL whole blood (CellSearch)**				
**Baseline CTCs**	**N = 10**	**N = 10**	**N = 10**	**N = 30**
Mean (SD)	29.4 (55.2)	53.5(98.3)	16.6 (23.9)	33.2 (66.1)
Median (range)	10.3 (0.3–181.0)	5.1(0.8–291.3)	3.1 (1.3–72.8)	4.3 (0.3–291.3)
≥ 5 CTCs	5 (50%)	5 (50%)	4 (40%)	14 (47%)
**Week 12 CTCs**	***N*** **= 6**	***N*** **= 4**	***N*** **= 9**	***N*** **= 19**
Mean (SD)	8.5 (16.0)	1.3 (0.9)	0.7 (0.8)	3.3 (9.2)
Median (range)	0.9 (0.0–40.5)	1.0 (0.5–2.5)	0.0 (0.0–2.5)	0.8 (0.0–40.5)
≥ 5 CTCs	2 (33%)	0 (0%)	0 (0%)	2 (10%)
**Disease progression CTCs**	***N*** **= 7**	**N = 6**	***N*** **= 2**	***N*** **= 15**
Mean (SD)	62.6 (125.6)	62.3 (94.0)	0.3 (0.4)	54.1 (101.9)
Median (range)	*20.8 (0.5–345.3)*	28.4 (1.0–244.3)	0.3 (0.0–0.5)	3.3 (0.0–345.3)
≥ 5 CTCs	4 (57%)	3 (50%)	0 (0%)	7 (46%)
**CTC immune checkpoint biomarker expression at baseline**				
**PD-L1 Expression**				
≥ 1 CTC positive	6 (60%)	7 (70%)	4 (40%)	17 (57%)
≥ 50% CTCs positive	2 (20%)	3 (30%)	3 (30%)	8 (27%)
**PD-L2 expression**				
≥ 1 CTC positive	4 (40%)	2 (20%)	4 (40%)	10 (33%)
≥ 50% CTCs positive	2 (20%)	2 (20%)	3 (30%)	7 (23%)
**B7-H3 expression**				
≥ 1 CTC positive	8 (80%)	9 (90%)	9 (90%)	26 (87%)
≥ 50% CTCs positive	8 (80%)	9 (90%)	8 (80%)	25 (83%)
**CTLA-4 expression**				
≥ 1 CTC positive	2 (20%)	1 (10%)	1 (10%)	4 (13%)
≥ 50% CTCs positive	1 (10%)	1 (10%)	1 (10%)	3 (10%)

### Prostate cancer CTCs express PD-L1, PD-L2, and CTLA-4; most frequently express B7-H3

PD-L1 expression was detected (on at least 1 CTC) at baseline in 40% of men with mHSPC, 60% of men with mCRPC pre-ARSI, and 70% of men with mCRPC post-ARSI, with fewer men who had > 50% of CTCs expressing PD-L1 (20–30% of each cohort) (Fig. 4**a**). PD-L2 expression was detected (on at least 1 CTC) in 40% of men with mHSPC, 40% of men with mCRPC pre-ARSI, and 20% of men with mCRPC post-ARSI. Fewer men had > 50% of CTCs expressing PD-L2 (20–30% of each cohort) (Fig. 4**b**). CTLA-4 expression was detected in at least 1 CTC in 10% of men with mHSPC, 20% of men with mCRPC pre-ARSI, and 10% of men with mCRPC post-ARSI. Only 10% of men in each cohort had > 50% of CTCs expressing CTLA-4 at baseline (Fig. 4**d**).

**Fig. 3:**
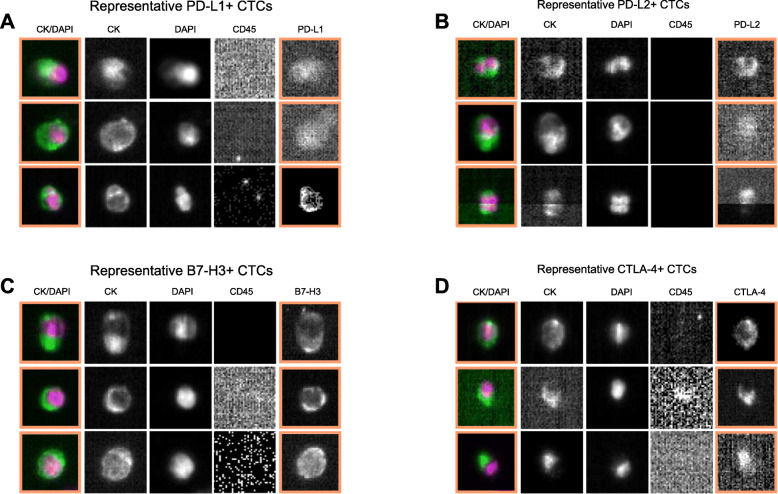
Representative patient CTCs stained for cytokeratin (CK), 4′,6-diamidino phenylindole (DAPI), CD45 (marker of leukocytes), and the immune checkpoint ligand of interest ([A] PD-L1, [B] PD-L2, [C] B7-H3, or [D] CLTA-4). CTCs are defined as being CK-positive, DAPI-positive, and CD45-negative. Orange boxes are indicators from the CellTracks Analyzer II that a cell was identified as marker-positive

**Fig. 4:**
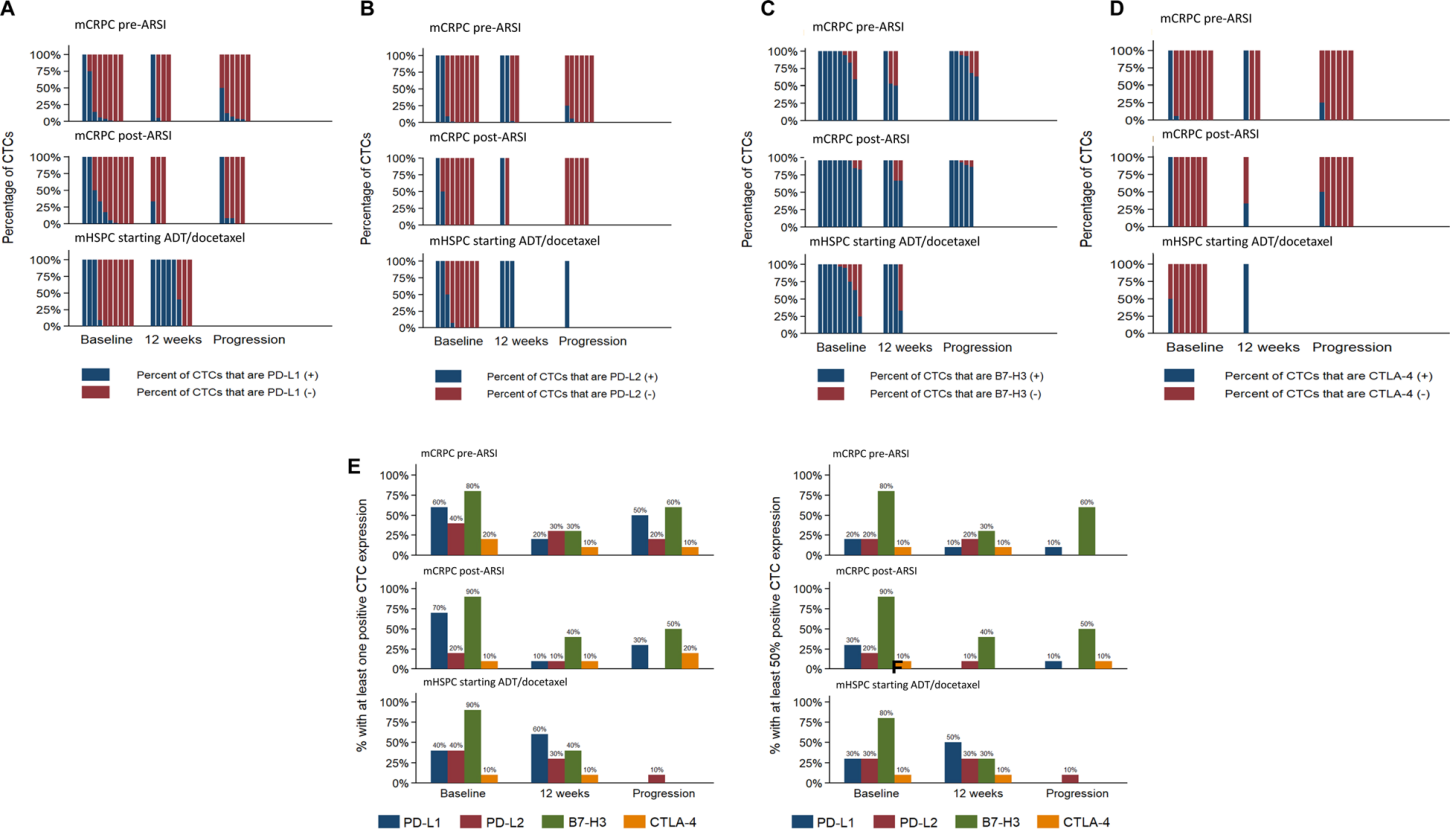
Expression of immune checkpoint biomarkers over time in each cohort. (A) PD-L1, (B) PD-L2, (C) B7-H3, (D) CTLA-4. Histograms made to depict percentage of total CTCs from each patient. Percentage of patients in each cohort with at least one marker-positive CTC at each timepoint (E). Percentage of patients in each cohort with at least 50% marker-positive CTCs at each timepoint (F). ARSI: Androgen receptor signaling inhibitors

Identified CTCs were characterized for expression of PD-L1, PD-L2, B7-H3, and CTLA-4 and representative positive CTCs and negative CTCs for each checkpoint ligand are shown (positive CTCs: Fig. 3; negative CTCs: **Supplemental Fig.** **2**). At baseline, B7-H3 was the most prevalent immune checkpoint ligand expressed (Fig. 4**c**). B7-H3 was present (detected on at least 1 CTC) in 90% of men with mHSPC, 80% of men with mCRPC pre-ARSI, and 90% of men with mCRPC post-ARSI. Across all cohorts, over half of the detected CTCs were positive for B7-H3 expression in 80% of men with mCRPC pre-ARSI, 90% of men with mCRPC post-ARSI, and 80% of men with mHSPC (Fig. 4**c**).

Among all cohorts, the rate of men with > 50% of CTCs positive for B7-H3 was 83%, PD-L1 was 27%, PD-L2 was 23%, and CTLA-4 was 10% (Table 2, Fig. 4). Because CTCs within each cohort and within each patient had heterogeneous expression of the tested immune checkpoints, reporting the proportion of men within the cohort shows the prevalence of expression in each disease setting, and reporting men with > 50% CTCs expressing each immune checkpoint represents the heterogeneity of the CTC populations for each patient.

### Expression of immune checkpoint ligands changes over time

In men with mCRPC pre-ARSI and mCRPC post-ARSI, the overall percentage of PD-L1+ CTCs appeared to decrease over time (Fig. 4**a**). In men with mCRPC pre-ARSI, the percentage of PD-L2+ CTCs decreased at progression compared to baseline and 12 weeks on treatment (Fig. 4**b**). None of the men with mCRPC post-ARSI who had detectable CTCs at progression had any PD-L2+ CTCs (Fig. 4**b**). From baseline to 12 weeks and at progression, the percentage of B7-H3+ CTCs remained high in most men across all three cohorts (Fig. 4**c**). CTLA-4 expression remained low in men across all three cohorts with only one patient per cohort with CTLA-4+ CTCs (Fig. 4**d**).

### Men with mHSPC have more PD-L1-positive CTCs over time

In men with mHSPC treated with ADT/docetaxel, the percentage of PD-L1-positive CTCs increased from baseline (mean CTCs 17, 3 men with 100% PD-L1 positive CTCs) to 12 weeks (mean CTCs 0.7, 5 men with 100% PD-L1 positive CTCs) (Fig. 4**a**) despite declining CTC numbers. The proportion of men with mHSPC with any PD-L1+ CTCs increased from baseline (4 of 10 men) to 12 weeks (6 of 8 men) (Fig. 4**e**) in the absence of disease progression to the mCRPC disease state.

In men with mCRPC pre-ARSI, PD-L1 expression decreased over time during treatment with androgen signaling inhibitors (Fig. 4**e**). Among mCRPC men in pre-ARSI and post-ARSI cohorts, 60 and 70% of men, respectively, had at least one PD-L1+ CTC at baseline (Fig. 4**e**), while only 20 and 30% of men, respectively, had at least 50% PD-L1+ CTCs (Table 2**,** Fig. 4**f**). In mCRPC (for both men in pre-ARSI and post-ARSI settings), men with at least 1 PD-L1+ CTC or > 50% PD-L1+ CTCs decreased over subsequent treatment, at the week 12 timepoint (Fig. 4). Notably, one patient with mCRPC post-ARSI had 5 out of 5 CTCs positive for PD-L1 and 2 out of 3 CTCs positive for B7-H3 at baseline. Upon subsequent treatment with a PD-1 inhibitor, with initial disease stabilization followed by progression, CTCs emerged at progression that were PD-L1 negative but continued to express B7-H3 (**Supplemental Fig.** **3**).

### B7-H3 expression is prevalent on prostate cancer CTCs

B7-H3 expression was prevalent throughout all cohorts and timepoints. Men with mCRPC pre-ARSI had mean 29 ± 55 CTCs at baseline, 8.5 ± 16 CTCs at 12 weeks, and 63 ± 125 CTCs at progression (Table 2). Men with mCRPC post-ARSI had mean 54 ± 98 CTCs at baseline, 1.3 ± 0.9 CTCs at 12 weeks and 62.3 ± 94 CTCs at progression. Men with mHSPC had mean 16.6 ± 23.9 CTCs at baseline, 0.7 ± 0.8 CTCs at 12 weeks, and 0.3 ± 0.4 CTCs at progression. The majority of men in all cohorts and at each time point had > 50% B7-H3 expressing CTCs (Fig. 4**c**).

### PD-L2 and CTLA-4 expression remains relatively stable over time

PD-L2 expression on CTCs remained low but largely unchanged over time. In all three cohorts, the percentage of men with at least one PD-L2+ CTC decreased slightly across the three collection timepoints (Fig. 4**e**). However, the percentage of men with at least 50% PD-L2+ CTCs remained the same in men with mCRPC pre-ARSI and mHSPC and decreased from 20 to 10% in men with mCRPC post-ARSI (Fig. 4**f**). The percentage of men with at least 50% CTLA-4+ CTCs was 10% at baseline in all three cohorts (Fig. 4**f**).

At 12 weeks, 10% of men with mCRPC post-ARSI had at least one CTLA-4+ CTC but did not have > 50% CTCs expressing CTLA-4. Few men’ CTCs across all cohorts and collection time points expressed CTLA-4. Of note, 10% of men with mCRPC pre-ARSI and mHSPC had at least 50% CTLA-4+ CTCs and this proportion of men stayed low over duration of treatment and time of disease progression (Fig. 4).

## Discussion

A major goal of precision immuno-oncology in prostate cancer is to identify men most likely to respond to specific immunotherapy approaches such as ICI therapy [47]. In men with mCRPC, the objective response rate to single agent CTLA-4 or PD-1 blockade is low at 5–10% [8–11, 28], thus indicating a major unmet need for predictive biomarkers. These responding men frequently lack MSI detection and while genomic predictors such as tumor mutation burden (TMB) [48], *CDK12* loss [23], or *LRP1b* alterations [49] may identify some men who have extraordinary and durable responses to PD-1 blockade, most men with mCRPC who respond to ICI therapy lack identifiable biomarkers to explain this response. In this study, we have characterized four such immune checkpoint biomarkers on CTCs (PD-L1, PD-L2, B7-H3, and CTLA-4) from men with metastatic prostate cancer and described the clear heterogeneity of each IC ligand on CTCs across metastatic prostate cancer disease states and between and within men over time. CTLA-4 expressing CTCs were found at the pre-defined success threshold of 20% in only the mCRPC pre-ARSI cohort, not in men with mHSPC (10%) or men with mCRPC post-ARSI (10%). Otherwise the pre-defined success thresholds were met, with least 20% per cohort having PD-L1 and PD-L2 expressing CTCs, as well as at least 50% of each cohort having B7-H3-expressing CTCs.

The results of this study are consistent with our hypothesis that immune checkpoint ligands are expressed heterogeneously on prostate CTCs between disease settings, within cohorts, and between timepoints. While others have shown PD-L1 expression on the surface of CTCs in other solid tumors including prostate cancer [14, 15, 50–52], we have detected 4 different checkpoint ligands PD-L1, PD-L2, B7-H3, and CTLA-4 on CTCs. Furthermore, we can evaluate changes between disease states, among men in a given cohort, and between timepoints during a given patient’s clinical course. A critical observation of the present study is this heterogeneity, particularly of PD-L1 and PD-L2 expression on CTCs, where 10–20% of such men had a majority of CTCs expressing one of the markers of immune evasion and exhaustion. We attempted to describe this diversity of prostate cancer biology using these descriptors of any CTC with immune checkpoint ligand and > 50% of CTCs expressing the immune checkpoint ligand in any patient sample. We observed that men with mCRPC exhibited similar CTC immune checkpoint biomarker heterogeneity in enzalutamide/abiraterone acetate plus prednisone sensitive and resistant settings. Both mCRPC cohorts (pre-ARSI and post-ARSI) had fewer men with PD-L1+ CTCs from baseline to 12 weeks. This trend differed from the cohort of mHSPC men, whose percentage of men with PD-L1+ CTCs increased from baseline to 12 weeks. The percentage of mHSPC men with PD-L1+ CTCs at 12 weeks surpassed the number of mHSPC men with B7-H3+ CTCs. Such data could suggest a future targeted strategy of PD-1 pathway blockade in such men with abundant PD-L1/2 CTCs to test the predictive value of CTC PD-L1/2 detection.

In many men, only a subset of CTCs expressed a certain ligand at any one time point. For the first time, we have described CTLA-4 expression on CTCs in cancer, a checkpoint that is known to be expressed on immune cells and pituitary cells relevant to the known efficacy and toxicity of CTLA-4 blockade in men, and which has been reported to be expressed on some cancer cell lines [53, 54]. The observed heterogeneity of CTLA-4, PD-L1 and PD-L2 expression reinforces the notion that multiple distinct phenotypes of tumor cells often co-occur within a patient and that the isolation of metastatic CTCs from peripheral blood can help characterize tumor cell populations for therapeutic targeting better than a tissue biopsy that could exclude some subpopulations, especially those that are dynamic and whose expression changes longitudinally with treatment [55].

Currently US FDA-approved immunotherapies target PD-L1, PD-1 or CTLA-4; however, this study highlights the potential importance of B7-H3 as a target for immunotherapy. B7-H3 was almost universally expressed on CTCs in each disease cohort and at all timepoints. Given its high degree of expression on CTCs in men with prostate cancer and its correlation with poor clinical outcome [17–20], immunotherapies that utilize B7-H3 as a targeting mechanism might have success in upregulating endogenous T-cell response against aggressive tumors. The persistent expression of B7-H3 expressing CTCs with loss of PD-L1 expression on CTCs in a man with mCRPC treated with a PD-1 inhibitor immediately suggests a possible mechanism of immune evasion and future targeting.

There are several limitations to this current study. These include the small sample size of each cohort, limited clinical outcomes, and the unclear prognostic or predictive significance of identifying CTCs expressing these immune checkpoints as it pertains to immune checkpoint inhibition in the same men. Our patients were not often treated with ICIs in the mCRPC setting given the lack of FDA approvals and lack of consistent efficacy of ICIs in this setting, and therefore we cannot determine if these CTCs with immune checkpoint expression specifically change for any ICI therapy. However, this preliminary study shows the feasibility of characterizing immune checkpoints expression on CTCs and tracking these immune checkpoint-expressing CTCs over time. This finding forms the basis for future therapeutic studies embedding these CTC biomarkers in prospective trials to predict response and to monitor immune checkpoint expression over time.

This study reveals the heterogeneity of immune checkpoint expression on CTCs in men with metastatic prostate cancer and suggests a promising future exists for the development of CTC characterization assays that could be used clinically as real-time biomarkers of disease burden and treatment response. Our findings suggest that immune checkpoint inhibitors may have the potential to benefit some but not all men with metastatic prostate cancer. Future studies with larger patient populations could show stronger correlations between immune checkpoint ligand expression and treatment response. With the addition of future studies assessing this relationship between immune checkpoint expression and response, CTC characterization of peripheral blood has the potential to serve as a rapid, noninvasive prognostic or monitoring tool for the use of immune checkpoint inhibitors in the treatment of prostate cancers.

## Supplementary Information

**Additional file 1 Supplemental Fig. 1.** Consort Diagram Some men did not have detectable CTCs in every sample at a given time point. As a result, the number of samples analyzed for a particular biomarker may be less than the total number of men assessed at that timepoint. **(A)** mCRPC pre-ARSI cohort: 10 men had samples collected at baseline, 6 men at 12 weeks on treatment, and 7 men at progression, **(B)** mCRPC post-ARSI cohort: 10 men had samples collected at baseline, 4 men at 12 weeks on treatment, and 6 men at progression, **(C)** mHSPC ADT/docetaxel cohort: 10 men had samples collected at baseline, 9 men at 12 weeks on treatment, and 2 men at progression.

**Additional file 2 Supplemental Fig. 2.** Representative examples of patient CTCs that were negative for the immune checkpoint ligand of interest ([A] PD-L1, [B] PD-L2, [C] B7-H3, or [D] CLTA-4).

**Additional file 3 Supplemental Fig. 3.** Patient with mCRPC post-ARSI cohort, who had CTCs expressing PD-L1 at baseline and marker positivity disappeared during and after treatment with PD-1 inhibitor (A). CTCs expressing B7-H3 were not captured at baseline but captured at 12-weeks on treatment and progression with PD-1 inhibitor (B).
