# Supplementary figures and images for: Expression of immune checkpoints on circulating tumor cells in men with metastatic prostate cancer
Source: Biomark Res. 2021 Feb 18;9:14. doi: 10.1186/s40364-021-00267-y (PMC7890610; doi:10.1186/s40364-021-00267-y)

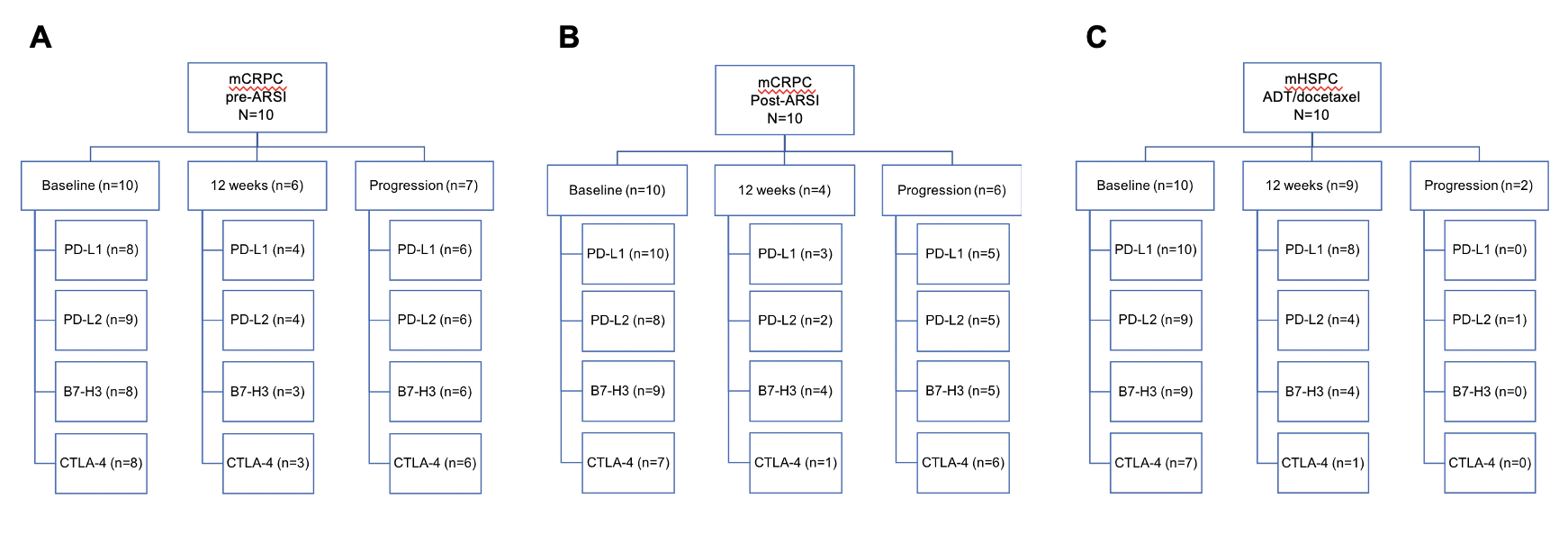

Supplement: Supplementary file 1 — Additional file 1 Supplemental Fig. 1. Consort Diagram Some men did not have detectable CTCs in every sample at a given time point. As a result, the number of samples analyzed for a particular biomarker may be less than the total number of men assessed at that timepoint. (A) mCRPC pre-ARSI cohort: 10 men had samples collected at baseline, 6 men at 12 weeks on treatment, and 7 men at progression, (B) mCRPC post-ARSI cohort: 10 men had samples collected at baseline, 4 men at 12 weeks on treatment, and 6 men at progression, (C) mHSPC ADT/docetaxel cohort: 10 men had samples collected at baseline, 9 men at 12 weeks on treatment, and 2 men at progression. [file 40364_2021_267_MOESM1_ESM.png]

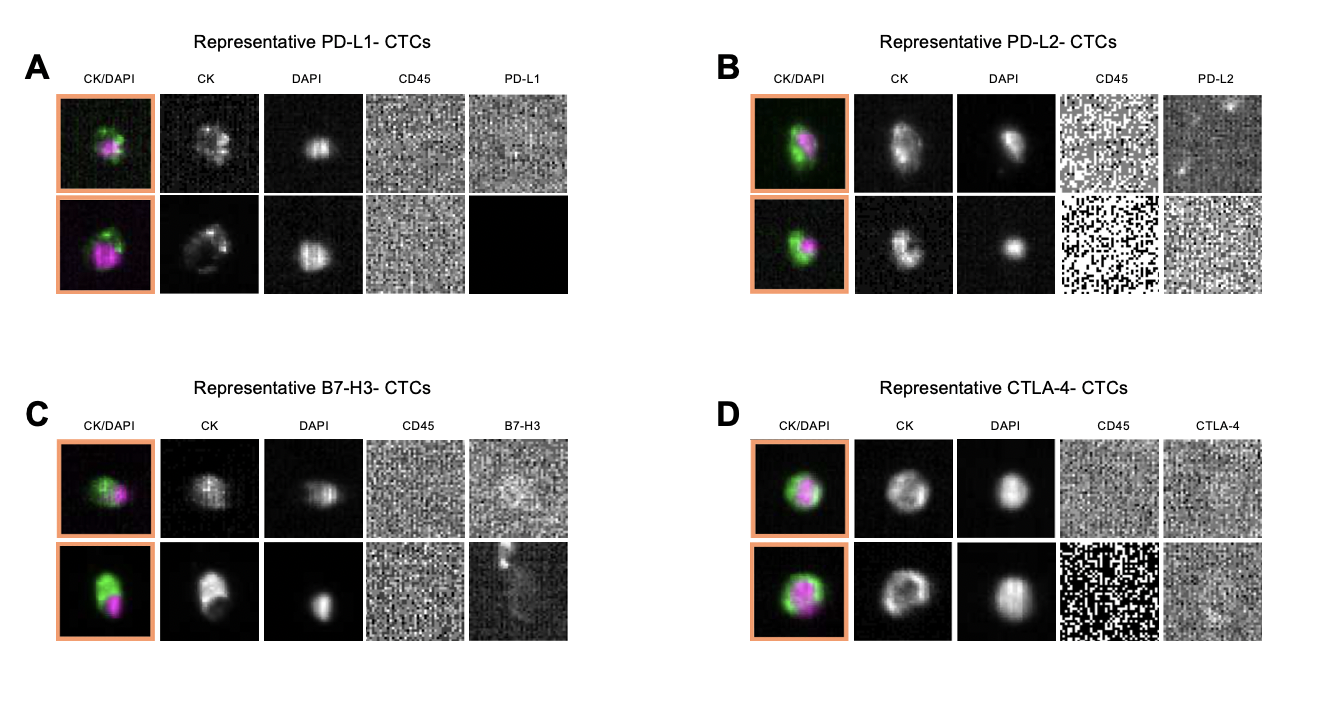

Supplement: Supplementary file 2 — Additional file 2 Supplemental Fig. 2. Representative examples of patient CTCs that were negative for the immune checkpoint ligand of interest ([A] PD-L1, [B] PD-L2, [C] B7-H3, or [D] CLTA-4). [file 40364_2021_267_MOESM2_ESM.png]

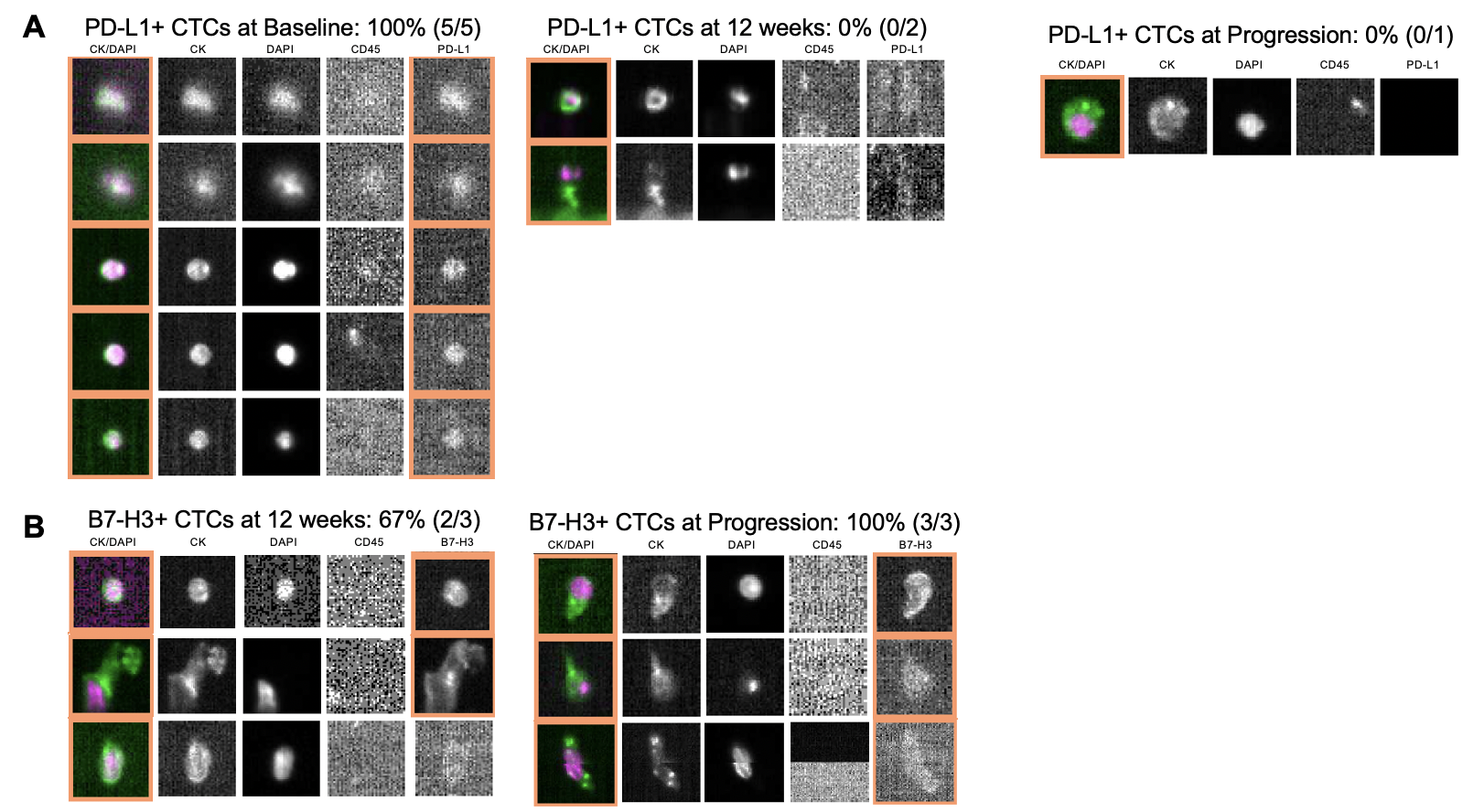

Supplement: Supplementary file 3 — Additional file 3 Supplemental Fig. 3. Patient with mCRPC post-ARSI cohort, who had CTCs expressing PD-L1 at baseline and marker positivity disappeared during and after treatment with PD-1 inhibitor (A). CTCs expressing B7-H3 were not captured at baseline but captured at 12-weeks on treatment and progression with PD-1 inhibitor (B). [file 40364_2021_267_MOESM3_ESM.png]
